# Adzuki Bean Peptide Prevents DSS-Induced Colitis by Modulating the Gut Microbiota

**DOI:** 10.3390/nu18182975

**Published:** 2026-09-11

**Authors:** Hang Zhou, Qun Shen, Liyang Wu, Zhihao Ma, Xin Ren, Jilite Wang, Zhihui Hao, Qingyu Zhao

**Affiliations:** 1 College of Food Science and Nutritional Engineering, China Agricultural University, Beijing 100083, China; 2 College of Food and Health, Beijing Technology and Business University, Beijing 100048, China; 3 College of Agriculture and Food Science, Hetao College, Bayannur 015000, China; 4 College of Veterinary Medicine, China Agricultural University, Beijing 100193, China

**Keywords:** adzuki bean, peptides, ulcerative colitis, gut microbiota, short-chain fatty acids

## Abstract

**Background:** The incidence of ulcerative colitis (UC) continues to rise, and food-derived bioactive peptides have attracted increasing attention as potential nutritional interventions for intestinal inflammation. This study investigated the preventive effects of the adzuki bean-derived peptide IFNNDPNNHP (IP10 peptide) on dextran sulfate sodium (DSS)-induced acute colitis in mice and explored the involvement of the gut microbiota. **Methods:** Mice received prophylactic IP10 peptide at 100 or 200 mg·kg^−1^. Colitis severity was assessed based on body weight, disease activity index, colon length, histopathology, and inflammatory cytokine levels. Gut microbiota composition was analyzed by 16S rRNA gene sequencing, and fecal short-chain fatty acid (SCFA) levels were quantified. Antibiotic-mediated microbiota depletion and fecal microbiota transplantation (FMT) were used to further evaluate the role of the gut microbiota. **Results:** Prophylactic IP10 peptide administration attenuated DSS-induced colitis, with the 200 mg·kg^−1^ dose more effectively attenuating body weight loss and colon shortening, reducing disease activity index scores, and modulating inflammatory cytokine levels. IP10 peptide increased microbial richness and diversity, decreased the relative abundance of *Escherichia-Shigella*, and increased the relative abundances of SCFA-associated taxa, including *norank_f__Muribaculaceae* and *Lachnospiraceae_NK4A136_group*. It also increased fecal SCFA levels, particularly acetate and butyrate; the high dose additionally restored propionate and valerate. When the gut microbiota was depleted using antibiotics, the protective effects of the IP10 peptide were no longer detectable, whereas FMT using fecal microbiota from donors receiving the high dose of IP10 peptide attenuated colitis and increased fecal SCFA levels in recipient mice. **Conclusions:** IP10 peptide alleviates DSS-induced acute colitis, and this effect may be partly associated with alterations in the gut microbiota, including the enrichment of SCFA-associated taxa and increased fecal SCFA levels. These findings support its potential as a functional food ingredient for intestinal health.

## 1. Introduction

Ulcerative colitis (UC) is a chronic inflammatory disease of the colon, affecting approximately 5 million people worldwide [1]. Recent epidemiological studies indicate that the incidence of ulcerative colitis continues to rise rapidly in regions such as Asia and Latin America [2]. Despite the expanding range of treatment options, some patients still struggle to achieve sustained remission. There are no significant sex differences in the onset of ulcerative colitis, with the peak incidence occurring between the ages of 30 and 40. As this age range coincides with the prime of life, the disease severely impacts both physical and mental health as well as social development. Its pathogenesis is multifactorial, primarily attributed to genetic and environmental factors, followed by disruptions in the gut microbiota, defects in the epithelial barrier, dysregulated immune responses, and psychological stress. Although the exact cause of UC remains unclear, it is generally believed to result from a disruption in the balance between the gut microbiota and the mucosal immune system. Treatment for UC is primarily pharmacological; mesalazine is the first-line treatment for mild to moderate active UC, while corticosteroids are the treatment of choice for moderate to severe UC flare-ups. However, due to their side effects and the body’s tolerance to them, corticosteroids are not suitable for long-term use. Compared with conventional pharmacological therapies, bioactive peptides have been reported to exhibit relatively low cytotoxicity and favorable biological activity, highlighting their potential for the prevention and management of UC. Examples include rice protein peptides [3], wheat peptides [4], yellow tea peptides [5], and corn peptides [6], all of which can alleviate inflammatory bowel disease.

Adzuki beans are a high-protein, low-fat food; their protein content (16.33–29.2%) is 2–3 times that of cereal crops, and they contain 18 amino acids, with glutamic acid being the predominant one. Studies have shown that glutamic acid can be used to alleviate acute colitis [7]. Adzuki beans are cultivated in more than 20 countries worldwide, with production concentrated primarily in East Asia. China is one of the world’s leading producers, with an estimated adzuki bean output of approximately 350,000 metric tons for the 2024/25 marketing year [8,9]. Ancient Chinese texts document the anti-inflammatory properties of adzuki beans, and numerous studies have confirmed their significant anti-inflammatory and antioxidant effects [10], all of which are closely related to the alleviation of colitis. A semi-solid-state enzymatically hydrolyzed adzuki bean protein hydrolysate rapidly increased serum antioxidant levels in mice 5 min after administration [11]. The adzuki bean peptide KQSESHFVDAQPEQQQR significantly inhibited the production of IL-1, IL-6, TNF-α, and MCP-1 in lipopolysaccharide (LPS)-induced RAW264.7 macrophages [12]. Durak et al. [13] found that alcohol-soluble protein peptides from adzuki beans exhibit strong free radical scavenging activity, while peptides derived from globulin and gluten demonstrated strong iron and copper ion chelating abilities, respectively. Our previous studies found that adzuki beans and their protein hydrolysates can effectively alleviate high-fat diet-induced inflammation and intestinal mucosal barrier damage. Among these, the adzuki bean peptide IFNNDPNNHP (IP10 peptide) is one of the potential active peptides identified in our preliminary screening of adzuki beans; however, its potential for preventing ulcerative colitis and the specific mechanisms involved remain unclear [14,15,16,17].

The gut microbiota, as a central microecosystem within the host’s intestine, plays a role in the digestion, absorption, and metabolism of nutrients. Studies have shown that an imbalance in the gut microbiota is closely associated with the onset and progression of UC [18]. This imbalance can promote intestinal inflammation and mucosal damage by altering the composition of gut bacteria, increasing the abundance of pathogenic bacteria, and disrupting the distribution of microbial metabolites. Short-chain fatty acids are important metabolites produced by the fermentation of gut microbiota, primarily including acetate, propionate, and butyrate, which serve as a vital energy source for colonic epithelial cells [19]. Existing research indicates that SCFAs are crucial for maintaining colonic homeostasis, and changes in their levels are closely associated with the severity of intestinal inflammation in UC [20,21]. A healthy gut microbiota regulates the expression of tight junction proteins by producing SCFAs, thereby maintaining the integrity of the intestinal barrier [22]. Studies have shown that oyster peptides protect against dextran sulfate sodium (DSS)-induced ulcerative colitis by increasing the abundance of *Faecalibaculum*, *Odoribacter*, and *Anaerostipes*, as well as the levels of acetate, propionate, and butyrate [23]. Similarly, peptides derived from large-leaf yellow tea alleviate ulcerative colitis by increasing the relative abundances of *Akkermansia* and *Lactobacillus*, while reducing the relative abundance of *Escherichia-Shigella*, and elevating levels of acetate, propionate, n-butyrate, and isobutyrate [5]. Therefore, reshaping the gut microbiota structure and its SCFA metabolic profile through peptide intervention may be an effective strategy for alleviating ulcerative colitis.

This study used a 2.5% DSS-induced acute colitis model to evaluate the preventive effects of the IP10 peptide, which was administered for 7 days before DSS exposure and continued during DSS administration. Disease severity was evaluated based on the disease activity index, colon length, colonic histopathology, and inflammatory cytokine levels. The effects of the IP10 peptide on gut microbiota composition and short-chain fatty acids (SCFAs) were also investigated. Furthermore, antibiotic (ABX) experiments and fecal microbiota transplantation (FMT) were conducted to determine whether the protective effects of the IP10 peptide depend on the gut microbiota. This study provides insights into the potential mechanisms underlying the preventive effects of the IP10 peptide against DSS-induced acute colitis.

## 2. Materials and Methods

### 2.1. Synthesis of the IP10 Peptide

The IP10 peptide was synthesized by Hefei Taikubio Co., Ltd. (Hefei, China), and its purity was determined to be greater than 95% by high-performance liquid chromatography and mass spectrometry.

### 2.2. Experimental Design

Male C57BL/6J mice (6 weeks old, weighing 22 ± 2 g) were purchased from Beijing Vital River Laboratory Animal Technology Co., Ltd. (Beijing, China). All mice were housed in a specific pathogen-free (SPF) environment (24 ± 2 °C, 55 ± 5% humidity, 12 h light/dark cycle) with unrestricted access to food and water. After body weights were recorded, mice were assigned random numbers using the RAND function in Microsoft Excel and allocated to groups with comparable mean body weights. A total of 64 mice were used in this study, and no animals died or were excluded from any of the experiments. All animal experiments were approved by the Animal Care Committee of China Agricultural University (AW10404202-2-1, AW40215202-5-04).

#### 2.2.1. Establishment and Management of the Ulcerative Colitis Model

Mice were acclimated in the SPF environment for 7 days, after which all mice were randomly divided into 4 groups (8 mice per group): the control group (Control), the DSS model group (DSS), the DSS + low-dose IP10 peptide group (IP10-L, 100 mg·kg^−1^), and DSS + high-dose IP10 peptide group (IP10-H, 200 mg·kg^−1^). The control and DSS model groups were administered saline via gavage, while the IP10 peptide groups were administered the corresponding dose of IP10 peptide via gavage for 14 days. Starting on day 7, mice in the model group and the IP10 peptide intervention groups were allowed to drink freely a 2.5% (*w*/*v*) DSS solution (Dalian Meilun Biotechnology Co., Ltd., Dalian, China) for 7 days to establish a model of ulcerative colitis; mice in the control group were allowed to freely drink sterile water. 

#### 2.2.2. Antibiotic-Mediated Gut Microbiota Depletion Experiment

After a 1-week acclimatization period, all mice were randomly divided into two groups (8 mice per group): the antibiotic + DSS group (ABX-DSS) and the antibiotic + DSS + IP10 peptide group (ABX-IP10, 200 mg·kg^−1^). Antibiotics were added to the drinking water of all mice (50 μg/mL vancomycin, 50 μg/mL streptomycin, 100 μg/mL ampicillin, 100 μg/mL neomycin, 100 μg/mL metronidazole, 100 μg/mL ceftazidime, 125 μg/mL ciprofloxacin, and 1000 μg/mL bacitracin) (Beijing Solarbio Science & Technology Co., Ltd., Beijing, China) for 14 days to induce gut microbiota depletion [24]. Subsequently, the ABX-DSS group was administered saline via daily oral gavage, while the ABX-IP10 group was administered a 200 mg·kg^−1^ IP10 peptide solution via daily oral gavage for 14 days. Starting on day 7, mice in all groups were allowed free access to 2.5% (*w*/*v*) DSS water for 7 consecutive days.

#### 2.2.3. Fecal Microbiota Transplantation Experiment

As in Section 2.2.1, mice in the DSS group and IP10-H group were designated as donor mice. Fecal samples were collected during the late intervention phase, placed in sterile centrifuge tubes, and stored at −80 °C. After a 1-week acclimatization period, recipient mice were randomly divided into two groups (8 mice per group): the FMT-DSS group and the FMT-IP10 group. Mice in the recipient groups were administered antibiotics in their drinking water for 14 days (as described in Section 2.2.2) to deplete the gut microbiota [24]. Subsequently, the recipient mice received 200 μL of fecal transplant solution daily for 15 consecutive days; starting on the seventh day of transplantation, ulcerative colitis was induced by adding 2.5% DSS to their drinking water. Fecal samples were processed according to previously reported methods [25].

Body weights were monitored daily throughout the experiment. On the final day, the disease activity index (DAI) was calculated based on body weight loss, stool consistency, and fecal blood presence, after which all mice were fasted for 12 h with free access to water, lightly anesthetized with pentobarbital, and euthanized by cervical dislocation. Feces and colon tissues were collected; colon length was measured; a portion of the colon tissue was fixed in 4% paraformaldehyde for histological analysis; another portion was homogenized for biochemical assays; and the remainder was rapidly frozen in liquid nitrogen and stored at −80 °C.

### 2.3. Disease Activity Index (DAI) and Histopathological Analysis of Colonic Tissue

The DAI was calculated as the sum of scores for three symptoms: weight loss, stool consistency, and bloody stools [26]. DAI assessment was not blinded. The collected colonic tissue from the mice was fixed in 4% paraformaldehyde solution, embedded in paraffin, sectioned, and stained with hematoxylin and eosin (H&E). The histopathological features were then examined under a microscope (Nikon Corporation, Tokyo, Japan). Histopathological scores were assigned based on crypt damage, tissue damage, and inflammatory cell infiltration using a previously described method with minor modifications [27], with detailed scoring criteria provided in Table S1. Histological scoring was performed by investigators blinded to group allocation.

### 2.4. Enzyme-Linked Immunosorbent Assay

ELISA (Jiangsu Meimian Industrial Co., Ltd., Yancheng City, China) was used to measure interleukin-1β (IL-1β), interleukin-6 (IL-6), tumor necrosis factor-α (TNF-α), and interleukin-10 (IL-10) in colonic tissue; concentrations were normalized based on total protein content determined by the BCA assay. Colon tissue samples from six mice were randomly selected from each group for ELISA analysis.

### 2.5. Quantitative Real-Time Polymerase Chain Reaction (qPCR)

RNA was extracted from colon tissue using an RNA extraction kit (Tiangen Biotech Co., Ltd., Beijing, China). Colon tissue samples from six mice were randomly selected from each group for qPCR analysis. After determining the concentration of RNA extracted from the colon tissue, cDNA was synthesized using a reverse transcription kit (Nanjing Vazyme Biotech Co., Ltd., Nanjing, China) and amplified on a PCR instrument (Roche Diagnostics, Basel, Switzerland) using Taq Pro Universal SYBR qPCR Master Mix (Nanjing Vazyme Biotech Co., Ltd., Nanjing, China). Relative mRNA expression levels of *Il1b*, *Il6*, *Tnf*, and *Il10* were normalized to *Gapdh*. The primer sequences are listed in Table S2. Relative gene expression was calculated using the 2^−ΔΔCt^ method.

### 2.6. Gut Microbiome Analysis

Total microbial DNA was extracted from 100 mg of each fecal sample. The V3-V4 region of the bacterial 16S rRNA gene was amplified using primers 338F (5′-ACTCCTACGGGAGGCAGCAG-3′) and 806R (5′-GGACTACHVGGGTWTCTAAT-3′), followed by paired-end 300 bp sequencing on the Illumina MiSeq platform (Shanghai Majorbio Bio-Pharm Technology Co., Ltd., Shanghai, China). Raw reads were quality-filtered and merged using fastp and FLASH, respectively, and subsequently denoised and chimera-filtered using DADA2 in QIIME 2 to generate amplicon sequence variants (ASVs). Taxonomy was assigned using the classify-sklearn Naive Bayes classifier against the SILVA 138 16S_bacteria database with a confidence threshold of 0.7. Alpha diversity was assessed using the Chao, ACE, Shannon, and Simpson indices, and differences among groups were analyzed by one-way ANOVA followed by Tukey’s multiple comparisons test. PCoA and NMDS were performed at the ASV level based on Bray–Curtis distances, and group differences in NMDS were evaluated by ANOSIM with 999 permutations. LEfSe was performed at the genus level with an LDA score > 3.0. Pairwise genus-level comparisons in Section 3 were performed using two-sided Wilcoxon rank-sum tests with false discovery rate (FDR) correction for multiple comparisons. Bioinformatic analyses were performed on the Majorbio Cloud Platform (https://cloud.majorbio.com).

### 2.7. Quantification of SCFAs

Following the method reported by Tian et al. [28] with minor modifications, 100 mg of mouse feces was accurately weighed, and 800 μL of distilled water, 200 μL of 50% sulfuric acid, and 1 mL of ether were added. An appropriate amount of zirconia grinding beads was then added, and the mixture was homogenized in a homogenizer. The mixture was then centrifuged at 12,000× *g* for 10 min. The supernatant was collected, dehydrated with anhydrous calcium chloride, and an internal standard, 2-ethylbutyrate (Shanghai Aladdin Biochemical Technology Co., Ltd., Shanghai, China), was added. After vortex mixing, the solution was filtered through a 0.22 μm nylon filter membrane and prepared for GC-FID analysis. Fecal samples from six mice were randomly selected from each group for SCFA quantification.

Analysis was performed using a gas chromatograph (GC-2010Plus; Shimadzu Corporation, Kyoto, Japan) equipped with a hydrogen flame ionization detector (FID) and a fused-silica capillary column (SH-Stabilwax-DA, 30 m × 0.32 mm × 0.25 μm). The chromatographic conditions were as follows: injector temperature 250 °C, detector temperature 250 °C, and carrier gas high-purity nitrogen (N_2_, 30 mL/min). Temperature program: initial temperature 60 °C, held for 4 min; then increased at a rate of 6 °C/min to 180 °C, held for 3 min. Data acquisition was performed using LabSolutions software (version 5.110; Shimadzu Corporation, Kyoto, Japan).

### 2.8. Statistical Analysis

GraphPad Prism version 8.0 (GraphPad Software, San Diego, CA, USA) was used for statistical analysis and graphing. Data normality was assessed using the Shapiro–Wilk test. Longitudinal body-weight data were analyzed using two-way repeated-measures ANOVA with the Geisser-Greenhouse correction, followed by Tukey’s multiple-comparisons test for comparisons among groups at individual time points. An unpaired t-test was used for comparisons between two groups. For comparisons among multiple groups, normally distributed data were analyzed using one-way ANOVA followed by Tukey’s multiple-comparisons test, whereas non-normally distributed data were analyzed using the Kruskal–Wallis test followed by Dunn’s multiple-comparisons test. A *p*-value < 0.05 was considered statistically significant, and significant differences were denoted by different letters.

## 3. Results

### 3.1. IP10 Peptide Prevents Symptoms of DSS-Induced Acute Ulcerative Colitis in Mice

**Figure 1:**
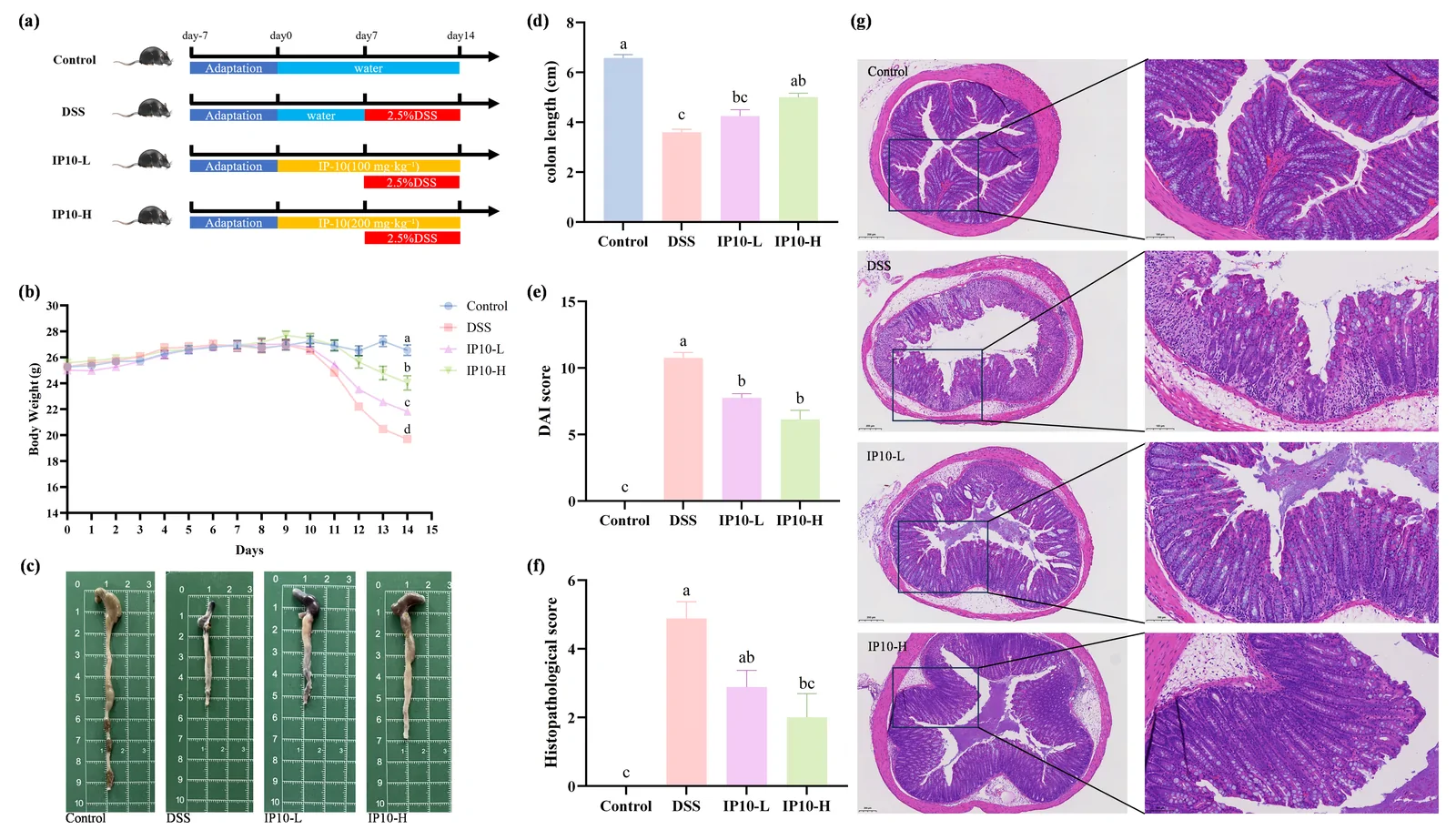
The IP10 peptide alleviates DSS-induced ulcerative colitis. Experimental design (**a**). Mouse body weight (**b**), representative colon photographs (**c**), colon length (**d**), disease activity index (**e**), and colon histopathological score (**f**). Histopathological changes in colon tissue from the experimental group observed via H&E staining (**g**) (scale bars: 200 μm, 100 μm). Control, normal control group; DSS, DSS-induced colitis model group; IP10-L, DSS model group receiving 100 mg·kg^−1^ IP10 peptide; IP10-H, DSS model group receiving 200 mg·kg^−1^ IP10 peptide. Different letters indicate a statistically significant difference at *p* < 0.05.

After 7 days of IP10 peptide pretreatment, there were no significant differences in body weight among the four groups (Figure 1b). Following the completion of pretreatment, DSS was administered; it was found that, starting on day 4, the DSS group exhibited a significant decrease in body weight compared with the control group, along with symptoms such as loose stools and bloody stools, demonstrating clear signs of colitis. At the conclusion of the experiment, compared with the control group, 2.5% DSS caused a significant shortening of colon tissue length in the DSS group (Figure 1c,d) and a significant increase in DAI scores (Figure 1e). Histopathological changes in the colon were examined using H&E staining (Figure 1f,g). The results showed that the colonic mucosal structure in the control group was intact, with regular crypt arrangement and abundant goblet cells, whereas DSS treatment led to marked epithelial disruption, crypt deformation, and inflammatory cell infiltration. In contrast, following IP10 peptide administration, the IP10-H group showed significantly less body-weight loss compared with the DSS group, attenuation of colon shortening, and lower DAI scores. High-dose IP10 peptide prophylactic treatment significantly reduced histopathological changes. Notably, the 200 mg·kg^−1^ IP10 peptide group showed greater effects than the 100 mg·kg^−1^ group on selected outcomes, particularly body-weight loss and DAI scores. In summary, the IP10 peptide significantly improved colonic mucosal damage and the degree of inflammatory cell infiltration in mice with colitis.

### 3.2. IP10 Peptide Inhibits DSS-Induced Colonic Inflammatory Response

**Figure 2:**
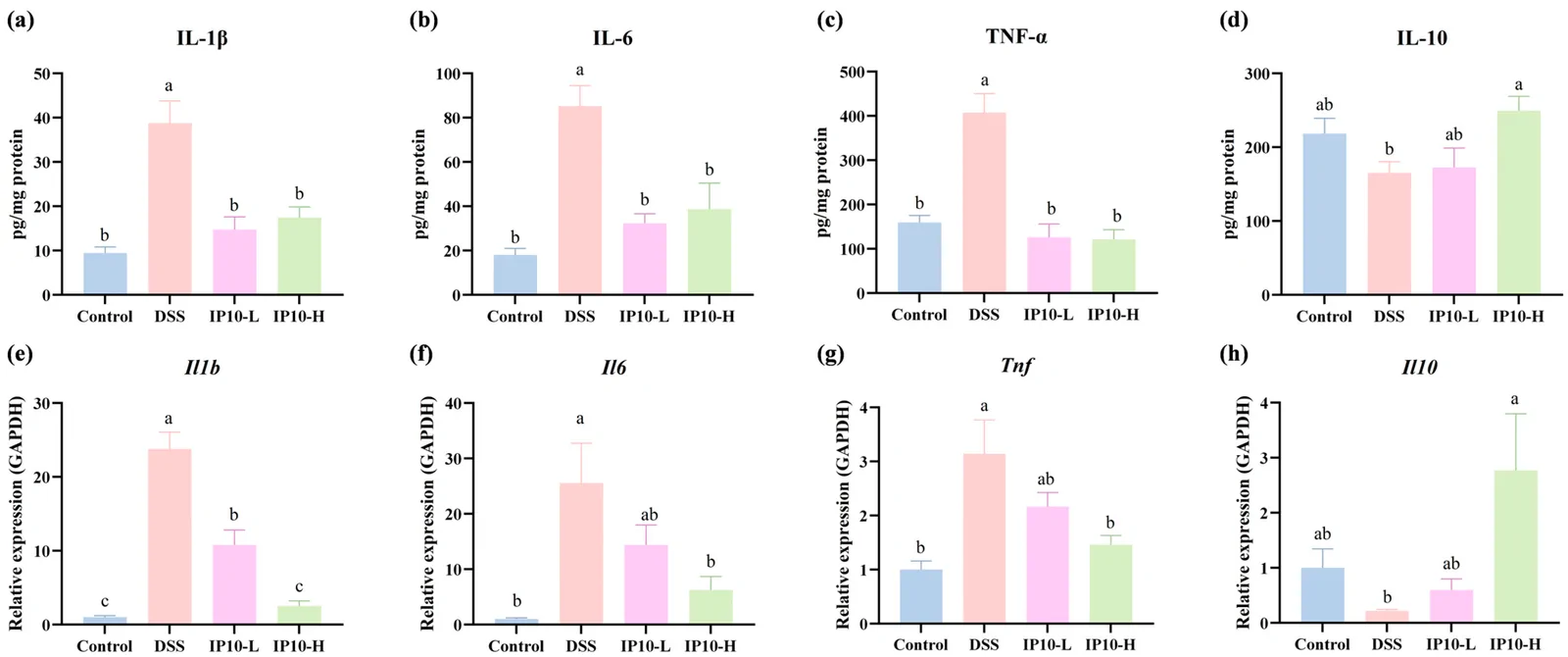
Effects of the IP10 peptide on inflammatory factors in DSS-induced ulcerative colitis. Protein levels of IL-1β, IL-6, TNF-α, and IL-10 in colonic tissue (**a**–**d**) and relative mRNA expression levels of the corresponding genes (**e**–**h**). *n* = 6. Control, normal control group; DSS, DSS-induced colitis model group; IP10-L, DSS model group receiving 100 mg·kg^−1^ IP10 peptide; IP10-H, DSS model group receiving 200 mg·kg^−1^ IP10 peptide. Different letters indicate a statistically significant difference at *p* < 0.05.

Compared with the control group, the protein expression levels of the pro-inflammatory cytokines IL-1β, IL-6, and TNF-α were all significantly elevated in the colonic tissues of mice in the DSS model group (Figure 2a–c, *p* < 0.05). Following prophylactic IP10 peptide administration, the levels of IL-1β, IL-6, and TNF-α in the 100 mg·kg^−1^ and 200 mg·kg^−1^ dose groups were both significantly lower than those in the DSS group (*p* < 0.05), and there was no significant difference between the two dose groups. As for the anti-inflammatory factor IL-10, its level was significantly higher in the 200 mg·kg^−1^ IP10 peptide-treated group compared with the DSS group, while there was no statistically significant difference between the 100 mg·kg^−1^ group and the DSS group (Figure 2d). This indicates that both doses of the IP10 peptide can suppress the DSS-induced elevation of pro-inflammatory factors, while the high dose of IP10 peptide exerts a more pronounced regulatory effect on IL-10. qPCR was then used to detect the mRNA expression of inflammation-related genes in colonic tissue. The results showed that in the DSS model group, the mRNA levels of *Il1b*, *Il6*, and *Tnf* were all significantly upregulated compared with the control group, while the mRNA level of *Il10* was downregulated compared with the control group (Figure 2e–h, *p* < 0.05). Both the 100 mg·kg^−1^ and 200 mg·kg^−1^ doses of the IP10 peptide significantly reduced *Il1b* mRNA expression; among these, the high-dose group showed significantly lower *Il6* and *Tnf* expression than the DSS group, whereas the reductions in the low-dose group did not reach statistical significance, while *Il10* levels were significantly elevated. These results indicate that low-dose IP10 peptide exhibits certain anti-inflammatory effects, while high-dose IP10 peptide effectively alleviates DSS-induced colitis in mice by dual inhibition of pro-inflammatory factors (IL-1β, IL-6, TNF-α) at both the genetic and protein levels and by increasing the expression of the anti-inflammatory factor IL-10, consistent with the observations of intestinal characteristics.

### 3.3. Effects of IP10 Peptide on the Composition of the Gut Microbiome in UC Mice

**Figure 3:**
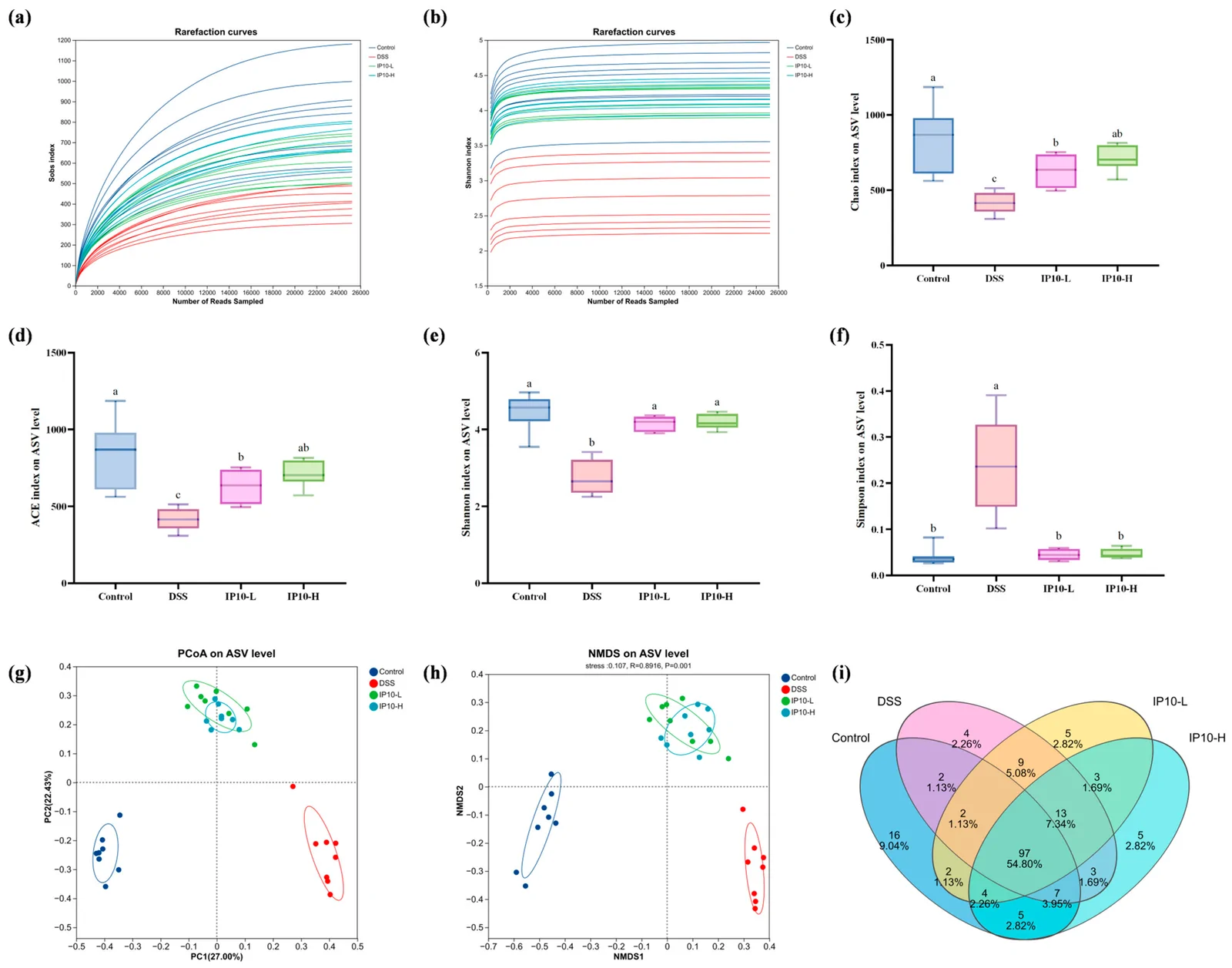
Effects of IP10 peptide on gut microbiota diversity in UC mice. Rarefaction curves of the Sobs index (**a**) and Shannon index (**b**); Chao index (**c**); ACE index (**d**); Shannon index (**e**); Simpson index (**f**); PCoA analysis (**g**); NMDS analysis (**h**); Venn diagram (**i**). *n* = 8. Control, normal control group; DSS, DSS-induced colitis model group; IP10-L, DSS model group receiving 100 mg·kg^−1^ IP10 peptide; IP10-H, DSS model group receiving 200 mg·kg^−1^ IP10 peptide. Different letters indicate a statistically significant difference at *p* < 0.05.

DSS-induced ulcerative colitis is accompanied by significant disruption of the gut microbiota [18]. To clarify the regulatory role of the IP10 peptide on the gut microbiome, 16S rRNA gene sequencing analysis was performed on fecal samples from mice in each group. The rarefaction curves for each sample gradually leveled off and eventually reached a steady state, indicating that the sequencing depth used in this study was appropriate and suitable for subsequent analysis of gut microbiota diversity and community structure (Figure 3a,b). α-diversity and β-diversity analyses were conducted to evaluate intragroup diversity and intergroup differences in community structure, respectively. The results showed that the richness (Chao and ACE indices) and Shannon index of the gut microbiota in the DSS model group were significantly lower than those in the control group, while the Simpson index was significantly higher (Figure 3c–f). This indicates that the DSS group exhibited lower microbial diversity and richness. In the IP10 peptide group, the Shannon index, Chao index, and ACE index were significantly higher than those in the DSS group, while the Simpson index was significantly lower. This indicates that the IP10 peptide can mitigate the decline in community richness and diversity in mice with DSS-induced ulcerative colitis. β-diversity analysis (PCoA, NMDS) showed that the DSS model group was clearly separated from the control group; DSS treatment caused a clear separation in gut microbiota composition from the control group, while the IP10 peptide group shifted overall toward the control group, indicating that IP10 peptide intervention could partially restore the altered gut microbiota structure (Figure 3g,h). Venn diagram analysis revealed that the four groups shared 97 ASVs, accounting for 54.80% of all detected ASVs, representing core microbial characteristics common to all groups (Figure 3i). The control and DSS groups had 16 and 4 unique ASVs, respectively. Following intervention with different doses of IP10 peptide, the community composition of each group exhibited a marked trend toward the control group, as evidenced by sharing more characteristic ASVs with the control group and reduced overlap with the DSS group. These results suggest that IP10 peptide intervention promotes the recovery of some microbiome characteristics associated with the Control group and shifts the microbiota composition away from the DSS-induced dysbiotic state. These findings are consistent with the trend toward partial recovery of community structure observed in the β-diversity analysis.

**Figure 4:**
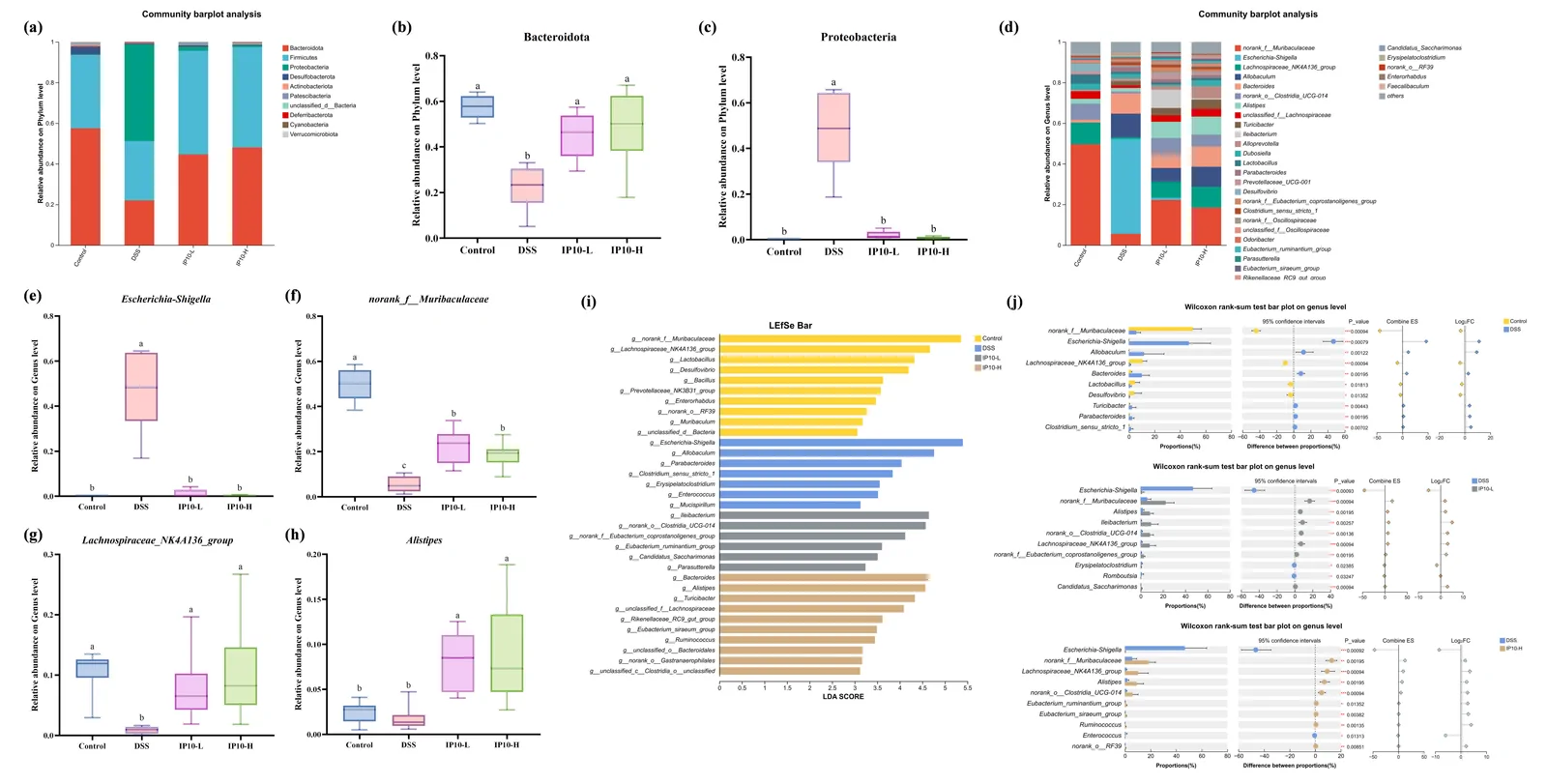
Effects of IP10 peptide intervention on the gut microbiota composition in UC mice. Gut microbiota composition at the phylum level (**a**), relative abundances of the phyla Bacteroidota (**b**) and Proteobacteria (**c**). Composition of the gut microbiota at the genus level (**d**); relative abundances of *Escherichia-Shigella* (**e**), *norank_f__Muribaculaceae* (**f**), *Lachnospiraceae_NK4A136_group* (**g**), and *Alistipes* (**h**) at the genus level. LEfSe analysis (**i**). Analysis of between-group differences in genus-level relative abundances of the gut microbiota (**j**): Control vs. DSS; DSS vs. IP10-L; DSS vs. IP10-H. Asterisks indicate statistical significance: * 0.01 < *p* ≤ 0.05, ** 0.001 < *p* ≤ 0.01, and *** *p* ≤ 0.001, *n* = 8. Control, normal control group; DSS, DSS-induced colitis model group; IP10-L, DSS model group receiving 100 mg·kg^−1^ IP10 peptide; IP10-H, DSS-induced colitis model group receiving 200 mg·kg^−1^ IP10 peptide. Different letters indicate a statistically significant difference at *p* < 0.05.

At the phylum level, the Circos plot (Figure S1a) visually illustrates the differences between groups. In the Control group, Bacteroidota and Firmicutes were the dominant phyla, forming the core framework of the gut microbiota. Compared with the Control group, DSS induction led to typical phylum-level dysbiosis in the mouse gut microbiota, with a significant decrease in Bacteroidota abundance and a significant increase in Proteobacteria abundance (Figure 4a–c). Both low-dose and high-dose IP10 peptide interventions effectively reversed this imbalance, with the relative abundance of Bacteroidota increasing, the relative abundance of Proteobacteria decreasing, and the microbial composition overall shifting toward that of the Control group. As shown in the LEfSe analysis, genus-level Circos plots, and heatmaps of the top 20 genera by abundance (Figure S1b–d), samples from the Control group clustered closely together, while those from the DSS group exhibited increased dispersion, the IP10 peptide intervention group, however, shifted back toward the Control group. Specifically, in the DSS model group, the relative abundance of Escherichia-Shigella increased significantly, while the relative abundances of *norank_f__Muribaculaceae* and *Lachnospiraceae_NK4A136_group* decreased significantly (Figure 4d–h, *p* < 0.05). The IP10 peptide intervention effectively reversed these changes, reducing the relative abundance of *Escherichia-Shigella* while increasing the relative abundances of *norank_f__Muribaculaceae*, *Lachnospiraceae_NK4A136_group* and *Alistipes* (*p* < 0.05).

Further LEfSe analysis (Figure 4i, LDA > 3) identified distinct group-associated microbial signatures among the four experimental groups. Consistent with the trends described above, the DSS group was characterized by *Escherichia-Shigella*, *Allobaculum*, *Parabacteroides*, and *Clostridium sensu stricto 1*, among others, as signature bacterial communities, whereas the IP10-L group was enriched with *Ileibacterium*, *norank_o__Clostridia_UCG-014*, *norank_f__Eubacterium_coprostanoligenes_group* and others. The IP10-H group was enriched with *Bacteroides, Alistipes, and Turicibacter*, among others. Pairwise differential abundance analysis more directly demonstrated the microbiota changes associated with IP10 peptide intervention. In the comparative analysis between the two groups, the IP10-L and IP10-H groups showed significantly higher abundances of *norank_f__Muribaculaceae, Lachnospiraceae_NK4A136_group, Alistipes, and norank_o__Clostridia_UCG-014* compared with the DSS group (Figure 4j). Therefore, IP10 peptide intervention at different doses significantly counteracted the increase in the relative abundance of some bacterial taxa and the decrease in others, thereby partially reversing DSS-induced alterations in gut microbiota composition.

### 3.4. The IP10 Peptide Restores DSS-Induced Decreases in Fecal Short-Chain Fatty Acid Levels

**Figure 5:**
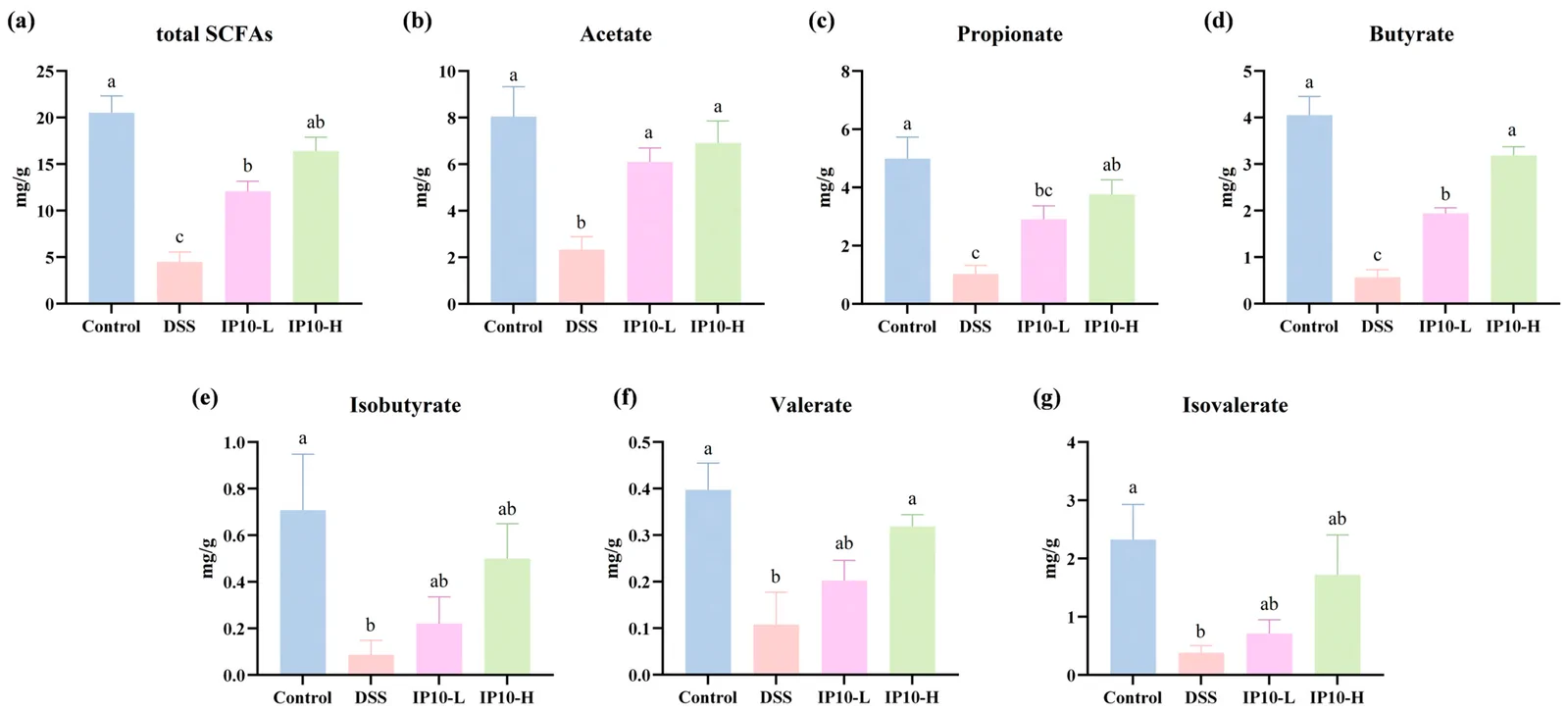
Effects of IP10 peptide on short-chain fatty acids in feces of UC mice. Total short-chain fatty acids (**a**), acetate (**b**), propionate (**c**), butyrate (**d**), isobutyrate (**e**), valerate (**f**), and isovalerate (**g**). *n* = 6. Control, normal control group; DSS, DSS-induced colitis model group; IP10-L, DSS-induced colitis model group receiving 100 mg·kg^−1^ IP10 peptide; IP10-H, DSS-induced colitis model group receiving 200 mg·kg^−1^ IP10 peptide. Different letters indicate a statistically significant difference at *p* < 0.05.

SCFAs are essential for strengthening the intestinal barrier and reducing intestinal inflammation. Numerous studies have shown that acetate, propionate, butyrate, and other SCFAs help maintain or improve intestinal epithelial barrier function in ulcerative colitis [29,30,31]. Our current study has found that the IP10 peptide regulates the homeostasis of the gut microbiota in UC mice and promotes the recovery of microbiota associated with SCFA production. Therefore, we further determined the levels of short-chain fatty acids in feces. Compared with the control group, the DSS group showed a significant reduction in total SCFAs in mouse feces (Figure 5a). Compared with the DSS group, total SCFA levels were significantly increased in all IP10 peptide intervention groups. Acetate and butyrate levels were significantly elevated in the IP10-L group, while concentrations of acetate, propionate, butyrate, and valerate were all significantly increased in the IP10-H group (Figure 5, *p* < 0.05). These results indicate that the IP10 peptide can effectively improve the levels of short-chain fatty acids in the feces of mice with ulcerative colitis.

### 3.5. Effects of the IP10 Peptide Under Antibiotic-Mediated Gut Microbiota Depletion

**Figure 6:**
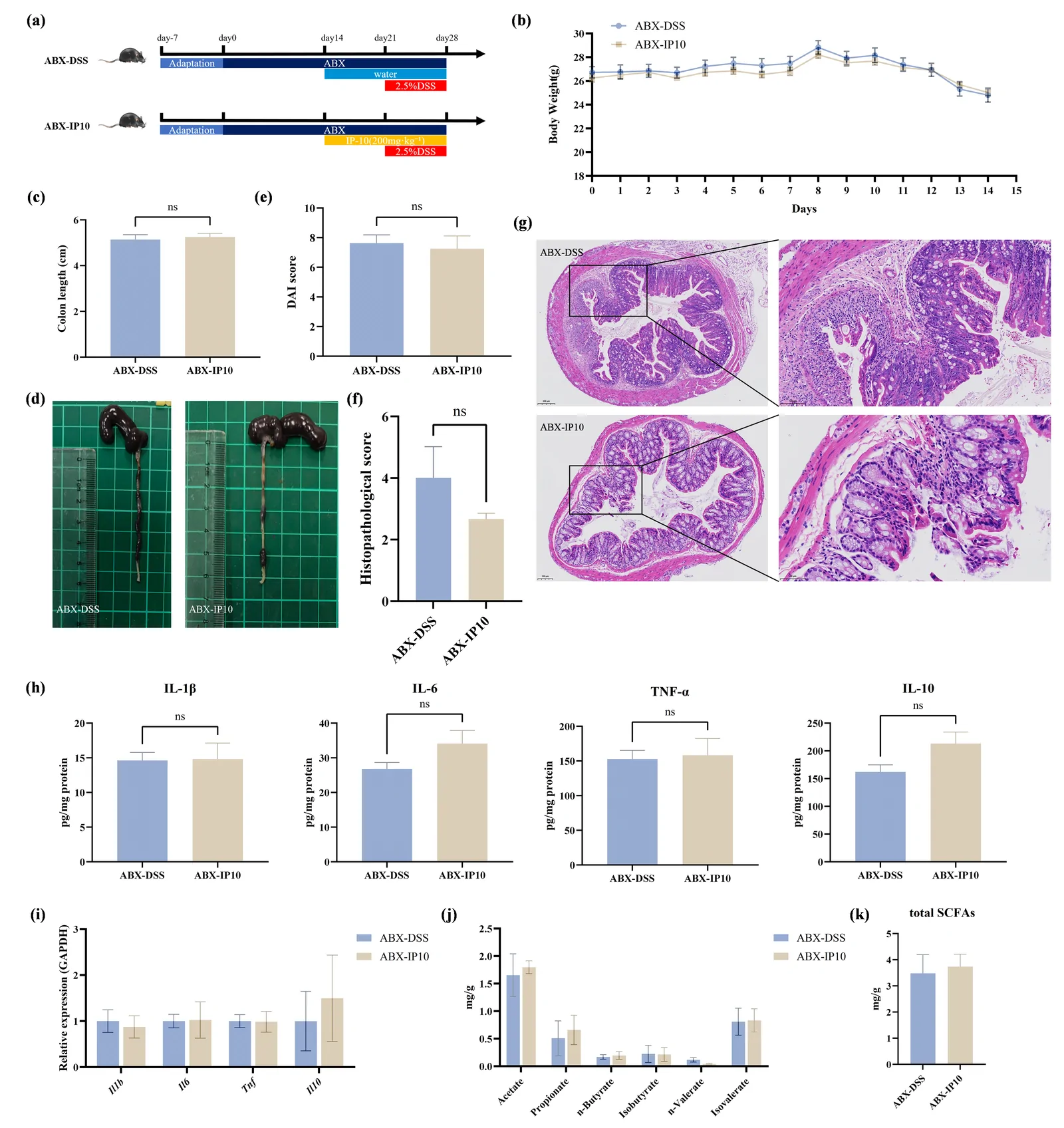
Effects of the IP10 peptide on DSS-induced colitis under antibiotic-mediated gut microbiota depletion. Experimental design (**a**). Mouse body weight (**b**), representative colon length (**c**), and colon photographs (**d**). Disease activity index (**e**) and colon tissue score (**f**); histopathological changes in colon tissue observed via H&E staining (**g**) (scale bars: 200 μm, 100 μm). Protein levels of inflammatory cytokines (**h**) and mRNA expression levels of related genes (**i**), levels of individual short-chain fatty acids (**j**), and total short-chain fatty acid content (**k**). Data are presented as mean ± standard error. ABX-DSS: DSS model group following antibiotic-induced gut microbiota depletion; ABX-IP10: DSS group receiving 200 mg·kg^−1^ IP10 peptide following microbiota depletion. ns indicates not significant.

We found that the IP10 peptide can reshape the gut microbiota, but it remains unclear whether the IP10 peptide prevents ulcerative colitis by regulating the microbiota. Therefore, we used ABX treatment to induce gut microbiota depletion in UC mice and to preliminarily assess the role of the gut microbiota (Figure 6a). Throughout the entire experimental period, the body weight change curves of the ABX-DSS and ABX-IP10 groups almost overlapped (Figure 6b). There were no significant differences in colon length or DAI scores between the two groups (Figure 6c–e). H&E staining showed that both groups exhibited mucosal damage and inflammatory cell infiltration, with no significant difference in histological scores (Figure 6f,g). Similarly, there were no significant differences in inflammatory cytokine levels—whether at the protein or gene level-between the ABX-IP10 group and the ABX-DSS group (Figure 6h,i). The levels of all six short-chain fatty acids and total short-chain fatty acids were low in both groups, with no significant differences between them (Figure 6j,k). Under the antibiotic-mediated microbiota-depletion conditions used in this experiment, no significant protective effect of the IP10 peptide was detected across these endpoints. These findings suggest that the gut microbiota plays an important role in the protective effects of the IP10 peptide observed in this study.

### 3.6. The Fecal Microbiota of IP10 Peptide-Treated Mice Exerts a Protective Effect Against Colitis

**Figure 7:**
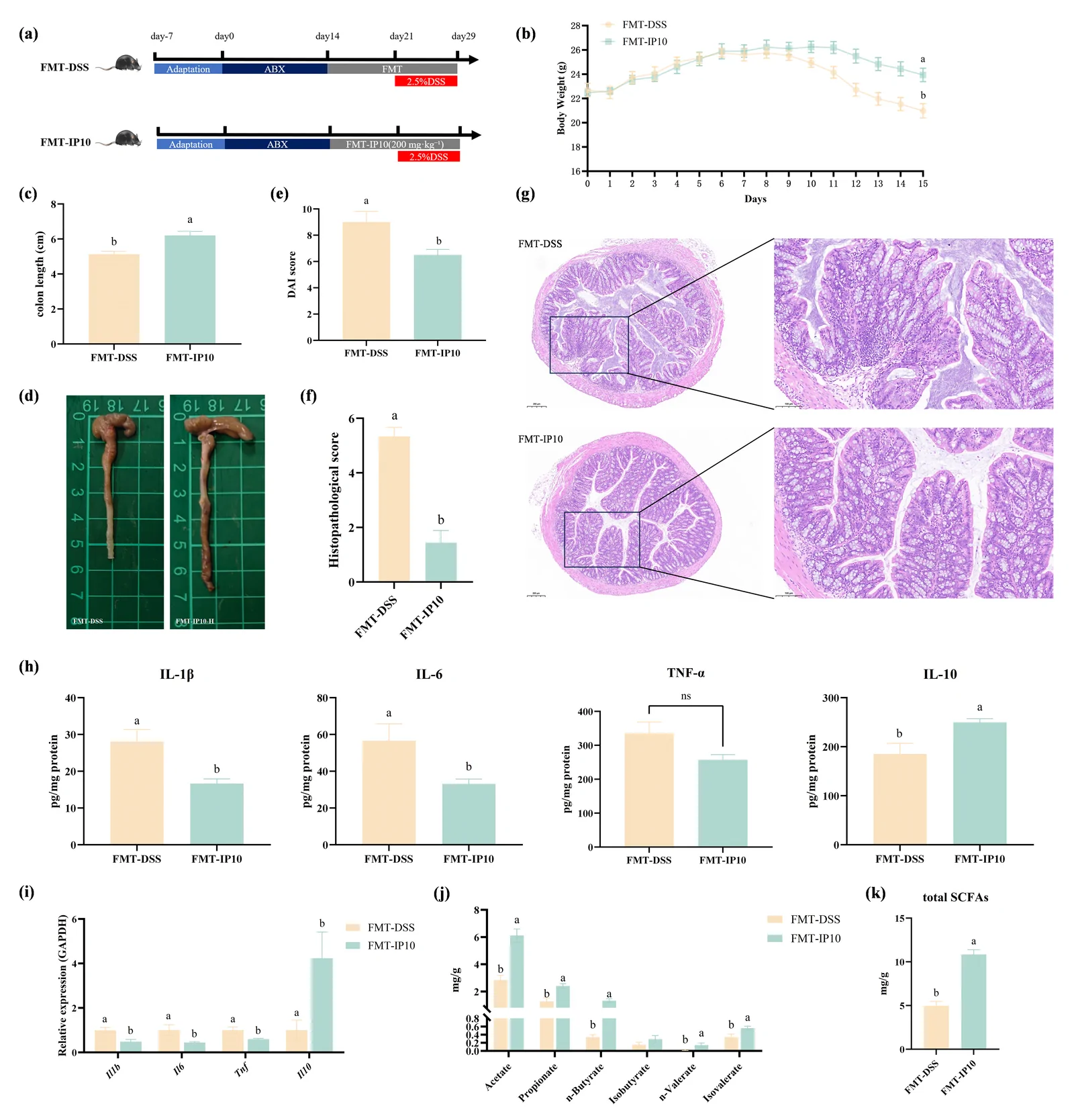
Effect of FMT on DSS-induced ulcerative colitis in mice. Experimental design (**a**). Mouse body weight (**b**), representative colon length (**c**), and colon photographs (**d**). Disease activity index (**e**) and colonic tissue score (**f**); histopathological changes in colonic tissue observed via H&E staining (**g**) (scale bars: 200 μm, 100 μm). Protein levels of inflammatory cytokines (**h**) and mRNA expression levels of related genes (**i**); levels of individual short-chain fatty acids (**j**) and total short-chain fatty acids (**k**). Data are presented as mean ± standard error. FMT-DSS: the DSS recipient group that received the fecal microbiota from the DSS donor group; FMT-IP10: the DSS recipient group that received the fecal microbiota from the IP10-H donor group. Different letters indicate a statistically significant difference at *p* < 0.05. ns indicates not significant.

To further verify whether the gut microbiota reshaped by IP10 peptide can mediate a protective effect against ulcerative colitis, we conducted fecal microbiota transplantation experiments (Figure 7a). Compared with recipient mice that received fecal microbiota from the DSS group (FMT-DSS), recipient mice that received fecal microbiota from the group receiving the IP10 peptide (FMT-IP10) exhibited significantly reduced colitis symptoms, as evidenced by a significantly smaller decrease in body weight (Figure 7b), significantly attenuated colon shortening, and a significant reduction in DAI scores (Figure 7c–e). Colonic histology also revealed that the colons of mice in the FMT-DSS group exhibited loss of crypt architecture and massive inflammatory cell infiltration. In contrast, the colonic tissue of mice in the FMT-IP10 group maintained a relatively intact epithelial barrier and crypt structure, with markedly reduced inflammatory infiltration in the submucosa and a significantly lower histological damage score compared with the FMT-DSS group (Figure 7f,g, *p* < 0.05). Analysis of inflammatory cytokine expression in colonic tissue (Figure 7h,i) revealed that the protein and mRNA expression levels of the pro-inflammatory cytokines IL-1β and IL-6 were significantly lower in the FMT-IP10 group compared with the FMT-DSS group (*p* < 0.05), *Tnf* mRNA expression was significantly suppressed and its protein levels showed a downward trend, while the protein and mRNA levels of the anti-inflammatory factor IL-10 were significantly elevated (*p* < 0.05), indicating that the gut microbiota established after IP10 peptide treatment can also suppress colonic inflammatory responses. We further measured SCFA levels in the feces of FMT recipient mice. Compared with the FMT-DSS group, the FMT-IP10 group showed significantly higher levels of acetate, propionate, butyrate, valerate, and isovalerate in mouse feces (Figure 7j), and the total SCFA content was also significantly increased (Figure 7k). This indicates that the gut microbiota reshaped by the IP10 peptide can also improve DSS-induced SCFA metabolic dysregulation and increase the fecal SCFA content, suggesting that SCFAs may be an important mediator through which the IP10 peptide exerts its preventive effect via the gut microbiota.

## 4. Discussion

UC is a chronic, relapsing inflammatory bowel disease whose etiology has not yet been fully elucidated; it likely involves complex interactions among genetic susceptibility, intestinal mucosal barrier dysfunction, environmental factors, and gut microbiota dysbiosis. With the acceleration of social and economic development, the incidence of UC is also on the rise [32], and bioactive peptides have attracted growing interest for their potential to modulate intestinal inflammation [33].

The IP10 peptide was identified from adzuki bean protein hydrolysate. In silico analyses predicted no toxicity or carcinogenicity for the IP10 peptide (Table S3). In addition, exposure to 25–800 μg/mL IP10 peptide for 24 h did not reduce HepG2 cell viability under the tested conditions (Supplementary Method S1 and Figure S2). However, these computational and in vitro findings alone are insufficient to establish the in vivo safety or tolerability of the IP10 peptide, and further safety evaluation is required. The amino acid sequence of the IP10 peptide is IFNNDPNNHP; its *N*-terminus contains the hydrophobic amino acids Ile-Phe, and the sequence includes two Pro and four Asn residues, with a pI value of 5.08 (Figure S3). Previous studies have shown that the biological effects of food-derived bioactive peptides are related to factors such as peptide chain length, amino acid composition, residue position, and gastrointestinal digestive stability [34]. Many peptides with anti-inflammatory activity are typically rich in hydrophobic amino acids, which are mainly concentrated at the *N*-terminus. Anti-inflammatory peptides such as MMLDF [35], similar to the IP10 peptide, are also hydrophobic at the *N*-terminus and carry an overall negative charge. The IPP and VPP [36] peptides, which have been proven to possess anti-inflammatory effects, are both rich in Pro; studies indicate that Pro-rich peptides have significant potential to exhibit anti-inflammatory activity [34]. Furthermore, the walnut-derived tripeptide LPF can alleviate DSS-induced colitis in mice and partially restore gut microbiota diversity as well as the relative abundances of *Lachnospiraceae* and *Ruminococcaceae* [37]. The IP10 peptide has a high content of Asn residues, accounting for 40% of the total amino acid residues. Soriano-Correa et al. [38] found that replacing the Asn residue in the middle of the anti-inflammatory tripeptide Cys-Asn-Ser (CNS) with Asp reduced the peptide’s anti-inflammatory activity. Asn residues may contribute to the biological activity of anti-inflammatory peptides by influencing their conformational stability, physicochemical properties, and chemical reactivity. The heptapeptide WFNNAGP derived from matsutake mushrooms contains a consecutive Asn-Asn structure; in a DSS-induced colitis model in mice, it alleviated weight loss, diarrhea, colon shortening, and histopathological damage, and improved oxidative stress and intestinal barrier function [39]. Furthermore, the soybean-derived decapeptide SLVNNDDRDS, containing 20% asparagine residues, exhibits anti-inflammatory effects in Caco-2 cells by inhibiting NF-κB-mediated inflammatory responses and downregulating the expression of pro-inflammatory genes [40]. Therefore, the high proportion of Asn residues in the IP10 peptide and their specific arrangement may contribute to its structural characteristics and biological activity.

At both doses of 100 mg·kg^−1^ and 200 mg·kg^−1^, the IP10 peptide demonstrated protective effects in improving weight loss, reducing the disease activity index, restoring colon length, and alleviating colonic mucosal damage and colonic inflammatory factors. The high dose showed greater effects across several endpoints in improving weight loss and the disease activity index. It should be noted that IP10 peptide administration was initiated 7 days before DSS exposure and continued during DSS challenge. Therefore, the present findings should be interpreted within a prophylactic rather than therapeutic context. Disruption of the gut microbiome is closely associated with the pathogenesis of inflammatory bowel disease [41]. It has been reported that food-derived peptides can significantly alter the structure of the gut microbiota [42,43]. The results from 16S rRNA sequencing showed that DSS treatment significantly reduced the richness and diversity of the gut microbiota in mice, leading to a severe imbalance in the microbial community structure, consistent with previous studies [44]. Following IP10 peptide intervention, α-diversity recovered markedly, and the microbial community structure reverted toward that of the control group. The Chao and ACE indices in the IP10-H group showed a recovery trend toward the control group, with a significantly greater degree of recovery than that observed in the IP10-L group. PCoA analysis showed that the DSS group was clearly separated from the Control group, while the IP10-L and IP10-H groups were clearly distinguished from the DSS group, indicating that IP10 peptide intervention reversed the DSS-induced shift in microbial community structure and partially restored the community structure. Venn diagrams further demonstrated that DSS treatment led to the loss of certain microbial features shared with the control group, whereas these features reappeared following IP10 peptide intervention, suggesting that the IP10 peptide may help restore the community composition disrupted by DSS treatment. The above results indicate that the protective effect of the IP10 peptide against colitis is closely related to the gut microbiota.

To clarify the role of the gut microbiota in the preventive effects of the IP10 peptide against ulcerative colitis, this study established an antibiotic-treated mouse model using combined antibiotic treatment and validated the findings through fecal microbiota transplantation experiments. Results from the antibiotic-mediated microbiota-depletion experiment showed that the beneficial effects of the IP10 peptide on weight loss, elevated disease activity index, colon shortening, histopathological damage, and pro-inflammatory cytokine expression were not evident under the conditions used. No significant differences were observed between the ABX-IP10 group and the ABX-DSS group, indicating that an intact gut microbiota plays an important role in the IP10 peptide to exert its preventive effects. However, because the extent of microbiota depletion was not quantitatively confirmed, the present findings do not exclude possible microbiota-independent effects. Furthermore, SCFA metabolism was disrupted in colitis-affected mice subjected to ABX intervention. An increasing number of studies have applied fecal microbiota transplantation to the treatment of Crohn’s disease (CD) and ulcerative colitis [45,46]. Results from fecal microbiota transplantation experiments showed that, compared with recipient mice that received donor microbiota from the DSS group, those that received microbiota from donor mice receiving 200 mg·kg^−1^ IP10 peptide exhibited reduced body weight loss, lower DAI scores, alleviated colonic shortening, and significantly reduced histopathological damage in the colon. At the same time, the expression of pro-inflammatory factors IL-1β and IL-6 in the colons of recipient mice was significantly downregulated, while the expression of the anti-inflammatory factor IL-10 was significantly upregulated. This indicates that the gut microbiota reshaped by the IP10 peptide continues to play a role in preventing ulcerative colitis. These results suggest that the IP10 peptide may serve as a potential functional food ingredient for colitis by modulating the gut microbiota.

Building on the established role of the gut microbiota, this study further analyzed changes in gut microbiota composition following IP10 peptide intervention. Compared with healthy control mice, IBD mice typically exhibit reduced gut microbial species richness and altered microbial community composition [42], while an increased relative abundance of Proteobacteria is frequently associated with gut dysbiosis [45]. The enrichment of Proteobacteria is also closely associated with intestinal inflammation and disruption of the mucosal barrier [19]. At the phylum level, DSS treatment similarly led to an increase in the relative abundance of Proteobacteria and a decrease in the relative abundance of Bacteroidota, whereas IP10 peptide intervention partially reversed these changes. Further analysis of microbial relative abundances at the genus level revealed that the DSS group was characterized by *Escherichia-Shigella*, *Allobaculum*, and *Parabacteroides*, among others, whereas the relative abundance of *Escherichia-Shigella* was significantly reduced following IP10 peptide intervention. An increased relative abundance of *Escherichia-Shigella* has frequently been observed in DSS-induced colitis models compared with healthy control mice and has been associated with gut microbiota dysbiosis [47,48,49]. At the same time, the IP10-L group was enriched with taxa such as *Ileibacterium*, *norank_o__Clostridia_UCG-014*, and *norank_f__Eubacterium_coprostanoligenes_group*. The functional role of the genus *Ileibacterium* in intestinal homeostasis remains incompletely characterized. However, a recent DSS colitis study demonstrated that *Ileibacterium valens* was predominantly enriched in both the control and Brevilin A-treated groups, whereas its abundance was markedly reduced in DSS-treated mice [50]. In addition, compared with the DSS group, the IP10-H group showed increased relative abundances of norank_f__Muribaculaceae, *Lachnospiraceae_NK4A136_group*, *Alistipes*, and *Ruminococcus*, together with a decreased relative abundance of *Escherichia-Shigella*. Given the important role of microbiota-derived short-chain fatty acids in intestinal homeostasis [21], these taxonomic changes were further considered in relation to SCFA metabolism. These findings indicate that IP10 peptide intervention altered multiple DSS-associated taxa and partially shifted the gut microbiota composition toward the pattern observed in the Control group.

Notably, IP10 peptide intervention promoted the recovery of various bacterial communities associated with butyrate production. Studies have found that *Lachnospiraceae_NK4A136_group* [30,51,52], *norank_f__Muribaculaceae* and *norank_f__Eubacterium_coprostanoligenes_group* are all involved in short-chain fatty acid production. Zhi et al. reported that *Lachnospiraceae_NK4A136_group* and the *norank_f__Eubacterium_coprostanoligenes_group* can produce short-chain fatty acids and promote the synthesis of secondary bile acids, which helps reduce intestinal inflammatory responses and improve intestinal permeability in high-fat-induced obese mice [53]. Butyrate, as the primary energy substrate for colonic epithelial cells, not only maintains the integrity of tight junctions and enhances intestinal barrier function but also exerts anti-inflammatory effects by regulating intestinal immune homeostasis [54,55]. These results suggest that the improvement of DSS-induced colitis following IP10 peptide intervention was associated with increased relative abundances of taxa such as *Lachnospiraceae_NK4A136_group* and *norank_f__Eubacterium_coprostanoligenes_group*, which have been linked to butyrate metabolism. In addition to butyrate-associated microbiota, the IP10 peptide also promoted the recovery of various microbiota associated with acetate and propionate metabolism. *Muribaculaceae* is an important carbohydrate-fermenting microbiota in the mouse gut, closely associated with acetate and propionate production [56]; in this study, the IP10 peptide significantly increased the relative abundance of *norank_f__Muribaculaceae*. *Alistipes* is also associated with SCFA metabolism in the gut [57]. *Alistipes* is an anaerobic bacterium primarily found in the gut microbiota of healthy humans. It participates in the propionate synthesis pathway and has also been confirmed as a producer of acetate; the SCFAs it produces can exert anti-inflammatory effects through various immunometabolic pathways. Dziarski et al. [58] found in a DSS-induced colitis mouse model that oral administration of *Alistipes finegoldii* significantly alleviated colonic inflammatory damage, and its protective effect was associated with the restoration of gut microbiota homeostasis. Therefore, the changes in the relative abundances of the aforementioned taxa may be associated with the observed increases in fecal acetate and propionate levels following IP10 peptide intervention.

Since the bacterial genera enriched by the IP10 peptide are associated with SCFA metabolism, we measured the levels of short-chain fatty acids in mouse feces. The results showed that, compared with the control group, DSS significantly reduced the total short-chain fatty acid content in feces, while IP10 peptide (100 mg·kg^−1^, 200 mg·kg^−1^) significantly restored the total short-chain fatty acid content in the feces of mice with ulcerative colitis; specifically, following high-dose IP10 peptide intervention, the levels of acetate, propionate, butyrate, and valerate in the feces all rebounded significantly. Following antibiotic treatment, short-chain fatty acid levels in the feces of both the ABX-DSS and ABX-IP10 groups decreased substantially. This may be due to antibiotic-mediated gut microbiota depletion, which significantly reduced fecal SCFA levels and inhibited the production of relevant SCFAs; furthermore, there were no significant differences in the levels of individual short-chain fatty acids between the two groups. Fecal microbiota transplantation experiments further demonstrated that the levels of acetate, propionate, butyrate, valerate, and isovalerate in the feces of recipient mice that received IP10 peptide donor microbiota also significantly increased (*p* < 0.05). Butyrate provides energy to intestinal epithelial cells, promotes cell differentiation and proliferation, repairs the mucosal barrier, and enhances the expression of tight junction proteins [22]. It also acts as a histone deacetylase inhibitor to promote the differentiation of regulatory T cells, inhibit the activation of the NF-κB signaling pathway, downregulate the expression of pro-inflammatory factors such as TNF-α, IL-1β, and IL-6 [20], and alleviate ulcerative colitis by modulating the Nrf2/GPX4 signaling pathway [59]. Furthermore, IP10 peptide intervention did not significantly alter isobutyrate levels in the DSS group, as isobutyrate primarily originates from the fermentation of proteins and branched-chain amino acids, suggesting that the regulation of microbial metabolism by the IP10 peptide exhibits a certain degree of specificity. Therefore, short-chain fatty acids, particularly butyrate, may serve as potential mediators through which the IP10 peptide exerts its preventive and protective effects via the microbiota. This finding is consistent with the results reported by Fei et al. [42], who demonstrated that the IRW (Ile-Arg-Trp) peptide alleviated DSS-induced colitis by enriching short-chain fatty acid-producing bacteria and increasing the abundance of acetate, propionate, and butyrate. This suggests that modulating the microbiota and short-chain fatty acids may be a common mechanism by which food-derived bioactive peptides intervene in colitis.

Several limitations and translational considerations should be acknowledged. The acute DSS-induced colitis model used in this study does not fully reproduce the chronic and relapsing nature of human IBD [60]. Gastrointestinal digestion and absorption are important determinants of the bioavailability of food-derived peptides [61,62]; however, the gastrointestinal stability and bioavailability of the IP10 peptide were not evaluated in the present study and remain to be determined. Current clinical guidance recognizes dietary and nutritional management as part of IBD care, including during remission and maintenance phases [63,64]. Whether the IP10 peptide could serve as a nutritional adjunct in these settings requires further investigation.

## 5. Conclusions

This study demonstrates that the adzuki bean-derived IP10 peptide protects mice against DSS-induced acute colitis. Its protective effects are associated with reduced intestinal inflammation, restoration of SCFA-associated taxa such as *norank_f__Muribaculaceae* and *Lachnospiraceae_NK4A136_group*, and significantly increased fecal SCFA levels, particularly butyrate. Antibiotic-mediated microbiota depletion attenuated these benefits, whereas fecal microbiota from donors receiving the IP10 peptide partially transferred the protective phenotype to recipient mice, indicating that the gut microbiota plays an important mediating role. These findings provide a theoretical basis for developing IP10 peptide as a functional food ingredient for maintaining intestinal health, although its efficacy and safety in humans require further investigation.

## Data Availability

The original contributions presented in the study are included in the article/Supplementary Materials; further inquiries can be directed to the corresponding author.

## Supplementary Materials

The following supporting information can be downloaded at: https://www.mdpi.com/article/10.3390/nu18182975/s1, Method S1. Cell Viability Assay. Figure S1. Effects of IP10 peptide intervention on the gut microbiota composition of UC mice; Figure S2. Effect of IP10 peptide on HepG2 cell viability. Figure S3. Chemical structure of the peptide IFNNDPNNHP. Table S1. Histopathological scoring criteria for crypt damage, tissue damage, and inflammatory cell infiltration; Table S2. The primer sequences used in RT-qPCR; Table S3. Prediction of the IP10 Peptide. Reference [65] is cited in the Supplementary Materials.
