# Supplementary material for: Adzuki Bean Peptide Prevents DSS-Induced Colitis by Modulating the Gut Microbiota
Source: Nutrients. 2026 Sep 11;18(18):2975. doi: 10.3390/nu18182975 (PMC13610624; doi:10.3390/nu18182975)
Supplement: Supplementary file 1 [file nutrients-18-02975-s001.zip › nutrients-4538318-supplementary.pdf]

nutrients

MDPI

Supplementary Materials

Method S1. Cell Viability Assay

Cell viability was determined using a CCK-8 assay with minor modifications according to Dou et al. [65]. HepG2 cells were seeded in 96-well plates at 1×10^4 cells/well and cultured for 24 h. The cells were then treated with IP10 peptide (25, 50, 100, 200, 400, and 800 μg/mL) for 24 h. After treatment, 100 μL of DMEM containing 10% CCK-8 reagent was added to each well and incubated for 2 h. Absorbance was measured at 450 nm.

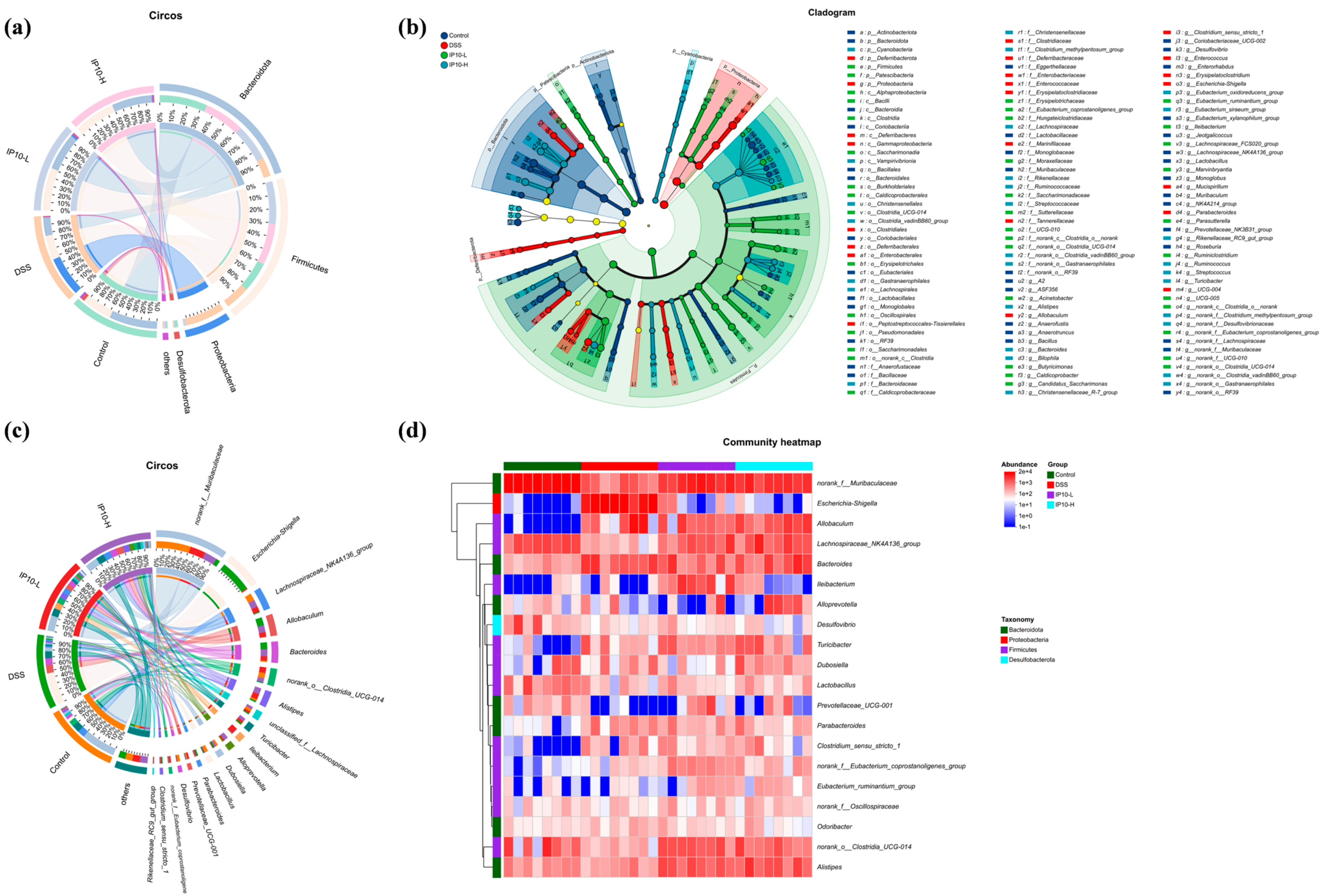

Figure S1. Effects of IP10 peptide intervention on the gut microbiota composition of UC mice.

Circos plots at the phylum level (a), LEfSe multilevel species hierarchy tree from phylum to genus (b), Circos plots at the genus level (c) and heatmap (d). n = 8. Control, normal control group; DSS, DSS-induced colitis model group; IP10-L, DSS-induced colitis model group treated with 100 mg·kg^{-1} IP10; IP10-H, DSS-induced colitis model group treated with 200 mg·kg^{-1} IP10.

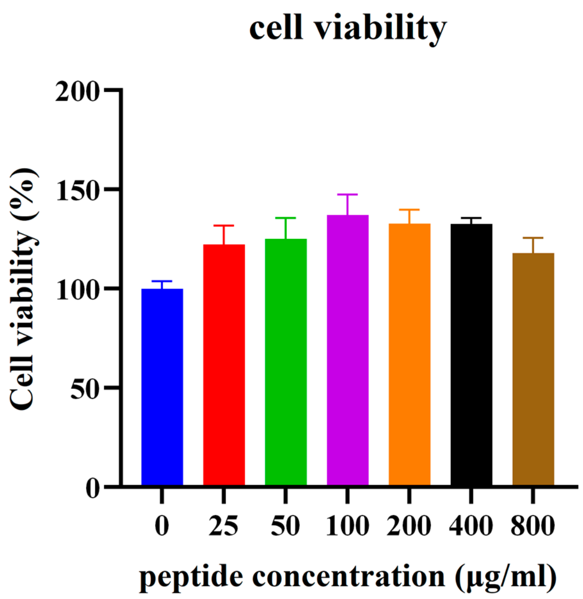

Figure S2. Effect of IP10 peptide on HepG2 cell viability
HepG2 cells were treated with IP10 peptide at concentrations of 25, 50, 100, 200, 400, and 800 μg/mL for 24 h, and cell viability was determined using the CCK-8 assay. Data are presented as the mean ± SEM.

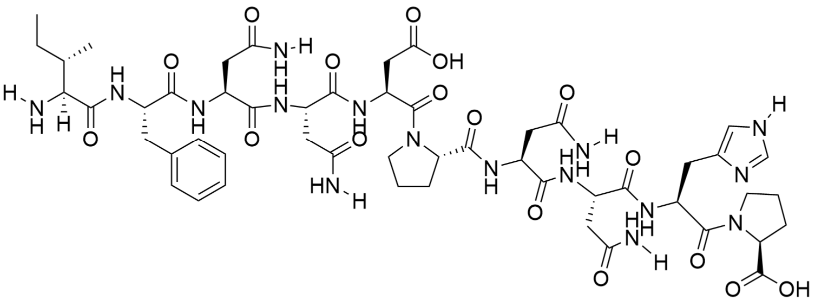

Figure S3. Chemical structure of the peptide IFNNDPNNHP

The chemical structure of the peptide was drawn using ChemDraw.

Table S1. Histopathological scoring criteria for crypt damage, tissue damage, and inflammatory cell infiltration.

Score	Crypt damage	Tissue damage	Inflammatory cell infiltration
0	None	None	None
1	1/3 basal damage	Mucosal damage	Mucosal infiltration
2	2/3 basal damage	Mucosal and submucosal damage	Mucosal and submucosal infiltration
3	Only surface epithelium intact	Transmural tissue damage	Transmural inflammatory infiltration
4	Complete loss of crypts and epithelium	—	—

Table S2. The primer sequences used in RT-qPCR.

Gene	Primer sequences (5′-3′)
Gapdh-F	GTGTTCCTACCCCCAATGTGT
Gapdh-R	ATTGTCATACCAGGAAATGAGCTT
Il1b-F	CAGGCAGGCAGTATCACTCATTG
Il1b-R	CGTCACACACCAGCAGGTTATC
Il6-F	TCCAGTTGCCTTCTTGGGAC
Il6-R	AGTCTCCTCTCCGGACTTGT
Tnf-F	TGGACTGTGGCATTGAGACAG
Tnf-R	CGACCCGTCCTTTGAATTTC
Il10-F	GCTCTTACTGACTGGCATGAG
Il10-R	CGCAGCTCTAGGAGCATGTG

Table S3. Prediction of the IP10 Peptide*.

Peptide Sequence	Isopotential point	Molecular weight/Da	Toxicity	Carcinogenicity	Hydrophobicity
IFNNDPNNHP	5.08	1181.23	Non-toxic	Non-carcinogenic	Hydrophilic

* The molecular weight, isoelectric point, and hydrophobicity of the IP10 peptide were predicted using ExPASy ProtParam (https://web.expasy.org/protparam/), and toxicity prediction for the peptide was performed using the Toxin Prep (https://webs.iiitd.edu.in/raghava/toxinpred/index.html) platform, based on the SVM (Swiss-Prot) algorithm, to predict the toxicity of the peptide. Carcinogenicity of the peptide was predicted using admet SAR (https://lmmd.ecust.edu.cn/admetsar2/).
